# Postpartum emergency department visits and readmissions in the 90 days after a singleton delivery

**DOI:** 10.1002/pmf2.70101

**Published:** 2025-09-10

**Authors:** Jecca R. Steinberg, Onyinyechukwu Ohamadike, Mahie Gopalka, Lynn M. Yee, Joe Feinglass

**Affiliations:** ^1^ Department of Obstetrics and Gynecology University of California San Francisco San Francisco California USA; ^2^ Department of Obstetrics and Gynecology Northwestern University Feinberg School of Medicine Chicago Illinois USA; ^3^ Department of Medicine Division of General Internal Medicine and Geriatrics Northwestern University Feinberg School of Medicine Chicago Illinois USA

**Keywords:** emergency department, hospital use, obstetrics, postpartum

## Abstract

**Introduction:**

Maternal morbidity and mortality are often concentrated in the first 90 days postpartum. Postpartum emergency department (ED) and hospital use provide valuable insight into peripartum health outcomes. We performed a health system‐wide analysis of sociodemographic, clinical, and hospital‐level factors associated with 90‐day postpartum ED visits and inpatient hospital admissions.

**Methods:**

This retrospective cohort study of all 90‐day postdelivery ED visits or inpatient admissions, referred to as “90‐day postpartum hospital use,” in a nine‐hospital Midwest health system included all births from January 2018 to June 2023. We applied a multilevel eco‐social framework to examine associations between factors within three domains and 90‐day postpartum hospital use. Exposure variables included sociodemographic factors (age, race/ethnicity, insurance, language, census zip code percent poor households), clinical factors (parity, clinical risk factors, body mass index [BMI], severe maternal morbidity during delivery admission [SMM], and other birth outcomes), and hospital type. We applied univariable and multivariable Poisson regression analyses.

**Results:**

Of 104,076 deliveries, 6879 (6.6%) were followed by 90‐day postpartum hospital use (ED visit *n* = 4559; hospital admission *n* = 2812). In bivariate analysis, all risk factors were significant. Chronic hypertension, substance use disorder, BMI ≥ 40 kg/m^2^, and SMM had the highest rates of 90‐day postpartum hospital use. After adjusting for all factors under investigation, Medicaid insurance (adjusted incidence rate ratio [aIRR] 1.43; 95% confidence interval [CI] 1.34–1.45), non‐Latinx Black race (aIRR 1.35; CI 1.25–1.46) SMM (aIRR 1.95; 95% CI 1.75–2.18), chronic hypertension (aIRR 1.59; CI 1.49–1.70), and BMI ≥ 40 kg/m^2^ (aIRR 1.52; CI 1.36–1.70) were associated with the greatest 90‐day postpartum hospital use risk compared to the reference categories. Primiparity and all other medical comorbidities had relatively weaker associations. Delivery at an exurban or community hospital was associated with greater 90‐day postpartum hospital use risk than delivery at the academic medical center.

**Conclusion:**

In this health system–wide analysis, 90‐day postpartum hospital use is associated with a combination of sociodemographic, clinical, and hospital factors, underscoring the need for a comprehensive mitigation strategy.

## 1 INTRODUCTION

Multiple studies document an increase in pregnancy‐related conditions and poor perinatal outcomes over the past two decades [1]. These trends track with advancing reproductive age and the rising prevalence of chronic conditions across the United States (US) reproductive population [1]. Physicians, policymakers, and health advocates have endeavored to improve health systems and obstetric outcomes. Most research focuses on prenatal or intrapartum care, with less attention on the postpartum period, even though it is well‐documented that postpartum morbidity is a major contributor to maternal mortality; Most maternal deaths occurr in the postpartum period [2].

Postpartum emergency department and hospital use reflects the limitations of postpartum outpatient care and provides valuable insight into opportunities to improve obstetric outcomes [3]. Our objective was to analyze 90‐day postpartum hospital use in a large, metropolitan‐area health system in the Midwest in order to identify demographic, clinical, and hospital risk factors. This study was undertaken to inform actionable interventions and improve postpartum care, particularly since many postpartum readmissions may be preventable.

## 2 MATERIALS AND METHODS

This retrospective cohort study used electronic health records and International Classification of Diseases—Version 10 (ICD‐10) diagnosis codes for all deliveries of Illinois residents from January 2018 to July 2023 in a metropolitan Chicago‐area health system. The health system includes a large academic medical center, three suburban hospitals, and five smaller, exurban community hospitals. We analyzed all singleton, live deliveries between 23 and 43 weeks gestational age and identified 90‐day post‐delivery emergency department visits or inpatient admissions to any system hospital, referred to as “90‐day postpartum hospital use”. We selected 90 days to reflect the “fourth trimester” during which obstetricians are the primary providers and patients continue to contend with morbidity risks from pregnancy [4]. The study was approved by the Northwestern University Institutional Review Board.

Sociodemographic risk factors included age, race/ethnicity, insurance, language preference, and census tract percent poor households. The following categories were used: (1) Patient age—≤19, 20–24, 25–29, 30–34, 35–39, and ≥40 years; (2) race/ethnicity (self‐reported) – non‐Latinx white, non‐Latinx Black, Latinx, Asian, and other/unknown including multiracial; (3) Insurance – Medicaid versus private or other, including Medicare disability; (4) Language—English versus non‐English; (5) Census tract percent poor households. Patient addresses were geocoded and matched to Illinois census tracts using 2021 5‐year American Community Survey data on the percent of families living at or below the poverty line, categorized as < 1%, 1%–2.99%, 3%–4.99%, 5%–7.99%, or 8% or greater poor households.

Clinical risk factors included parity, history of a prior cesarean birth, pre‐pregnancy hypertension, hypertensive disorders of pregnancy, history of depression or other serious mental illness (Table 1 footnotes), substance use disorder, pre‐gestational diabetes, gestational diabetes, anemia, any pre‐existing comorbidity (Table 1 footnotes), maternal body mass index in kg/m^2^ (BMI), and birth outcomes. Clinical risk factors were coded using ICD‐10 codes at delivery. BMI at delivery was classified as missing, < 25, 25–29.99, 30–34.99, 35–39.99 and > 40. Birth outcomes included severe maternal mortality (SMM) [5], vaginal birth complications, cesarean birth complications, neonatal intensive care unit admission, and preterm birth (< 37 weeks gestational age). Delivery specific complications were coded following methods of Asch et al. For vaginal births, these included laceration, hemorrhage, infection, and thrombosis. For cesarean births these included hemorrhage, infection, and operative and thrombotic complications [6].

**TABLE 1:** Characteristics of 90‐day post‐delivery discharge emergency department and inpatient admission visits between January 2018 and June 2023.

	All delivery admissions *n* = 104,076	Emergency department visit *n* = 4559	Inpatient hospital admission *n* = 2812	Any 90‐day postpartum hospital use *n* = 6899	Any 90‐day postpartum hospital use adjusted Incidence rate ratio (95% CI)^a^
**Percent all births**	100	4.4	2.7	6.6	
**Patient sociodemographic characteristics**					
**Maternal age (years)**					
≤ 19	1.1	8.9	2.7	11.2	1.18 (0.99‐1.40)
20–24	7.6	8.5	2.8	10.5	1.16 (1.07–1.26)
25–29	18.8	5.8	2.5	7.7	Reference
30–34	40.7	3.6	2.5	5.7	0.93 (0.87–0.99)
35–39	26.2	3.3	3.0	5.9	0.98 (0.91–1.05)
≥ 40	5.6	3.7	3.6	6.9	1.01 (0.90–1.13)
**Race/ethnicity (self‐reported)**					
Non‐Latinx White	59.7	4.0	2.6	6.1	Reference
Non‐Latinx Black	8.4	7.1	4.6	10.9	1.34 (1.24–1.45)
Latinx	14.9	5.7	2.5	7.7	1.07 (1.00–1.15)
Asian	6.2	2.8	1.9	4.3	0.89 (0.78–1.00)
Other/unknown	10.8	3.5	2.7	5.8	0.61 (0.25–1.46)
**Medicaid**	19.2	8.1	3.3	10.7	1.42 (1.33–1.51)
**Non‐English language**	5.1	5.4	2.4	7.4	1.17 (0.96–1.42)
**Census tract percent of poor households**					
<1	19.7	3.4	2.4	5.5	Reference
1–2.99	20.8	3.6	2.5	5.8	0.97 (0.91–1.04)
3–4.99	15.5	4.1	2.6	6.3	0.98 (0.91–1.05)
5–7.99	15.8	4.6	2.8	6.9	1.04 (0.97–1.11)
≥8.0	28.1	5.6	3.1	8.2	1.07 (0.99–1.15)
**Patient clinical characteristics**					
**Parity**					
Nulliparous	45.9	4.2	2.9	6.7	Reference
1	34.2	3.9	2.4	5.9	0.87 (0.82–0.92)
2 or more prior births	19.9	5.5	2.9	7.8	0.93 (0.87–0.99)
**Clinical Risk Factors**					
History of a prior cesarean birth	18.4	5.4	3.2	3.2	0.98 (0.91–1.06)
Pre‐existing hypertension	7.6	7.4	7.7	13.7	1.55 (1.44–1.66)
Hypertensive disorders of pregnancy	5.5	6.1	5.8	11.0	1.33 (1.23–1.45)
History of depression or other serious mental illness^b^	16.3	7.3	3.5	10.1	1.18 (1.10–1.25)
Substance use disorder	3.9	11.1	4.4	14.2	1.24 (1.14–1.35)
Pre‐gestational diabetes	3.5	8.5	5.5	12.6	1.20 (1.09–1.33)
Gestational diabetes	5.9	5.2	3.5	8.1	1.03 (0.95–1.11)
Anemia	11.5	5.8	3.3	8.5	1.09 (1.02–1.17)
Any pre‐existing comorbidity^c^	13.2	6.0	3.8	8.9	1.20 (1.13–1.28)
**Maternal Body Mass Index (BMI, kg/m^2^)**					
Missing^d^	8.0	4.2	2.5	6.3	1.13 (1.00–1.28)
<25	9.4	3.2	1.8	4.6	Reference
25–29.99	34.3	3.2	2.0	5.0	1.05 (0.95–1.16)
30–33.99	23.1	4.4	2.8	6.7	1.25 (1.13–1.39)
34–39.99	16.9	5.6	3.6	8.6	1.37 (1.23–1.52)
>40	16.3	8.2	4.9	12.0	1.53 (1.37–1.72)
**Birth Outcomes**					
Cesarean birth	28.2	5.6	3.6	8.5	1.26 (1.18–1.35)
Severe maternal morbidity	1.5	10.3	12.0	19.5	1.88 (1.68–2.11)
Vaginal birth complications^e^	14.6	5.4	3.1	8.0	1.35 (1.26–1.45)
Cesarean birth complications^f^	18.4	6.7	4.5	10.2	1.15 (1.05–1.26)
Neonatal Intensive Care Unit admission	7.7	5.7	4.4	9.3	1.18 (1.09–1.29)
Preterm birth	7.3	6.6	4.8	10.5	1.09 (1.01–1.19)
**Hospital‐Type**					
Academic Medical Center 1	60.0	3.0	2.5	5.2	Reference
Suburban Hospital 1	8.6	6.2	2.7	8.3	1.38 (1.29–1.48)
Suburban Hospital 2	14.0	5.2	3.1	7.7	1.56 (1.44–1.69)
Suburban Hospital 3	6.8	6.2	3.1	8.6	1.67 (1.53–1.82)
Five Exurban Community Hospitals 5	10.6	8.6	3.2	10.9	1.81 (1.68–1.94)

^a^ *p* < 0.001 for all comparisons except for non‐English language.

^b^ Other serious mental illness included ICD‐10 codes for schizophrenia, psychosis, and bipolar disorder.

^c^ Any pre‐existing comorbidity included cardiac disease, bleeding disorder, pulmonary hypertension, chronic renal disease, gastrointestinal disease, human immunodeficiency virus infection, and acquired immune deficiency syndrome, bariatric surgery, asthma, connective tissue or autoimmune disease, neuromuscular disease, thyrotoxicosis, neuromuscular disease.

^d^ 9.4% with missing BMI data.

^e^ Vaginal birth complications included laceration, hemorrhage, and all others (e.g., infectious and thrombotic complications) [6].

^f^ Cesarean birth complications included hemorrhage, infection, and all others (e.g., operative and thrombotic complications) [6].

Hospital characteristics included the hospital at which the delivery occurred categorized as: academic medical center, suburban hospital 1, suburban hospital 2, suburban hospital 3 and five exurban community hospitals.

We conducted descriptive analyses and chi square tests. We fit multivariable Poisson regression models to estimate the likelihood of 90‐day postpartum hospital use [7]. Standard errors were adjusted for patient identifiers indicating multiple births for the same individual. Patients from other states (3.4%) were excluded from the analysis.

To account for postpartum hospital visits at other hospitals outside of the hospital system 70 participants were randomly selected and their care‐everywhere external records were reviewed for emergency department visits and admissions to outside hospitals. All analyses were completed using Stata Version 17 software.

## 3 RESULTS

A total of 103,076 singleton deliveries for 84,725 unique patients occurred in the study period. Most deliveries occurred at the academic medical center (60%), followed by suburban hospitals (29.4%) and exurban community hospitals (10.6%; Table 1). In this cohort, 6899 (6.6%) deliveries were followed by 90‐day postpartum hospital use (ED visit *n* = 4559; hospital admission *n* = 2,812).

**FIGURE 1:**
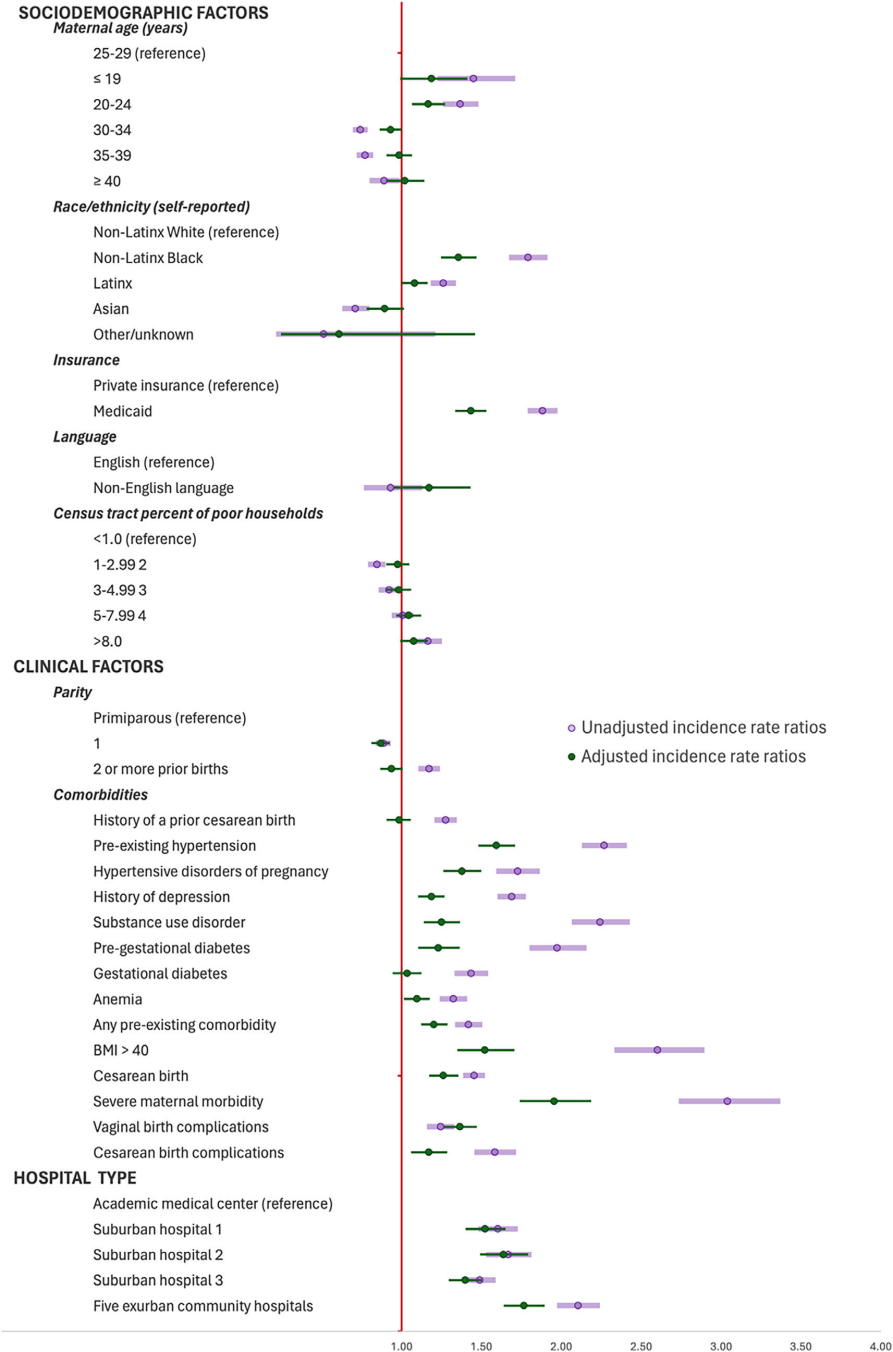
Unadjusted and adjusted incidence risk ratios of risk factors categorized as sociodemographic, clinical, and hospital factors. History of depression included other serious mental illness with ICD‐10 codes for schizophrenia, psychosis, and bipolar disorder. Vaginal birth complications included laceration, hemorrhage, and all others (e.g., infectious and thrombotic complications) [6]. Cesarean birth complications included hemorrhage, infection, and all others (e.g., operative and thrombotic complications) [6].

Regarding sociodemographic characteristics, there was an age gradient with younger patients having a higher 90‐day postpartum hospital use rate, however not all age differences were significant after adjusting for other covariates (Figure 1). Nearly 60% of the study population identified as non‐Latinx White, followed by Latinx (14.9%), Other/unknown (10.8%), non‐Latinx Black (8.4%) and Asian (6.2%). Non‐Latinx Black and other unknown race/ethnicity patients had significantly higher 90‐day postpartum hospital use rates compared to non‐Latinx white patients (Black aIRR 1.35, 95% CI 1.25–1.46). Medicaid covered nearly 20% of deliveries and was associated with a 42% higher likelihood of a 90‐day postpartum hospital use compared to private insurance (Medicaid aIRR 1.43, 95% CI 1.34–1.52). Differences by zip code poverty level were not significant after controlling for other factors.

Clinical factors had the strongest association with 90‐day postpartum hospital use. Patients with chronic hypertension were 53% more likely to have had a 90‐day postpartum hospital use (aIRR 1.59; CI 1.49–1.70). Almost 20% of patients with SMM had a 90‐day postpartum hospital use, with 88% higher adjusted risk, the highest of any risk factor studied (aIRR 1.95; 95% CI 1.75–2.18).

Regarding hospital type, patients who delivered at suburban and exurban community medical hospitals had significantly higher 90‐day postpartum hospital use rates than their peers at the academic medical center (Detailed hospital information in Table S1).

## 4 DISCUSSION

Postpartum hospital use remains relatively common, with 6.6% of individuals requiring ED care or hospital readmission within 90 days of delivery. Of all factors in the analysis, SMM had the highest association with 90‐day postpartum hospital use. However, given their prevalence, Medicaid insurance, racial minoritized status, cesarean birth, delivery complications, comorbid conditions (especially hypertension), and BMI > 40 had the most important associations with 90‐day postpartum hospital use. We present an updated investigation of postpartum hospital use in a Midwest location, including several years following the COVID pandemic. Despite a changing health landscape and significant investment in maternal health (e.g. postpartum Medicaid expansion), postpartum hospital use rates remain similar to those of previous studies of earlier timeframes, underscoring both the complexity and importance of this unique medical challenge. Our findings add nuance to sociodemographic risk factors by demonstrating no significant difference in 90‐day postpartum visits by patients’ neighborhood poverty levels. As system quality improvement is considered, focus should remain on the most relevant patient conditions rather than area poverty level.

This study reflects a largely affluent, insured population compared to the state‐wide population. However, significant racial and income disparities were still evident. Zip code poverty level differences were not independently significant after controlling for Medicaid and clinical risk factors, likely corresponding to the concentration of disease burden in higher poverty areas. Delivery at a suburban or exurban community hospitals had a greater association with 90‐day postpartum readmission than delivery at an academic medical center, which may be related to lesser availability of primary care, especially for Medicaid patients at these hospitals. Findings demonstrate that the drivers of postpartum readmission stem from both clinical and non‐clinical factors and a multifaceted, comprehensive approach will be needed to decrease postpartum readmissions.

Previous studies vary in geography, postpartum period, years, and risk factors evaluated. We found 4.4% and 2.7% of deliveries were followed by an ED‐visit or hospital admission, respectively. This aligns with studies conducted prior to 2020 across the US with similar rates of postpartum ED‐visits and inpatient admission [2, 8, 9]. Studies with a greater proportion of patients with Medicaid demonstrated more frequent of ED visits [10]. Across studies and in our findings, postpartum readmission follows well‐established trends, with low‐income communities and pregnant people of color bearing a disproportionate disease burden due to health care barriers, socioeconomic disparities, and systemic racism. Previous investigations predate 21^st^ century initiatives to improve maternal morbidity and mortality (e.g. the expansion of postpartum Medicaid coverage to one year postpartum). We examine later years, 2018–2023, but find little difference in established postpartum health patterns or socioeconomic and racial disparities.

A growing body of literature on hypertensive disorders of pregnancy demonstrates that patient‐tailored medication titration, patient education, remote patient‐led health monitoring, close outpatient virtual and in‐person primary care follow up may all reduce postpartum readmission [11]. These principles could be extrapolated to other chronic (e.g. diabetes, elevated BMI) or obstetric‐specific (e.g. SMM) conditions. Hospital‐based interventions including collaboration between obstetric and non‐obstetric primary care providers, coordinated discharge planning, and provider education are needed to complement individual clinical care. These interventions need to be implemented equitably to reach those enrolled in Medicaid and reduce disparities. Additionally, system‐wide policy shifts like expanded insurance coverage, state‐wide quality improvement collaboratives, and parental leave may improve postpartum health paradigms.

The study has several limitations. Analysis of postpartum hospital use through first listed ICD‐10 diagnosis codes resulted in uninformative data that that did not capture patients’ postpartum clinical conditions. While searches of interoperable records at other hospitals with the same electronic record vendor found almost no additional visits (only one of 70 randomly selected participants had a 90‐day postpartum hospital use at an outside hospital), we could not account for 90‐day postpartum hospital use outside of the electronic medical record system. The study focuses on one hospital system in the state of Illinois, with a relatively low percentage of Medicaid‐insured patients and may not be generalizable to other regions of the US.

## 5 CONCLUSION

Reducing 90‐day postpartum hospital use after discharge from the initial delivery requires a multifaceted approach. Our data bring a modern perspective to postpartum hospital readmission and help characterize the relative contributions of risk factors within a health system with a replicable analytic approach. Improvement in postpartum health and reduction in disparities will only be achieved by simultaneous clinical and social policy changes that address the root causes of peripartum inequality.

## CONFLICT OF INTEREST STATEMENT

The authors declare no conflicts of interest.

## CONSENT

This study was conducted with retrospective electronic medical record data with an institutional review board‐approved waiver of individual consent.

## Supporting information



Supporting Information
