# Supplementary material for: Postpartum emergency department visits and readmissions in the 90 days after a singleton delivery
Source: Pregnancy (Hoboken). 2025 Sep 10;1(5):e70101. doi: 10.1002/pmf2.70101 (PMC13344743; doi:10.1002/pmf2.70101)
Supplement: Supplementary file 1 [file PMF2-1-e70101-s001.docx]

Supplemental Appendix

*Postpartum emergency department visits and readmissions in the 90 days after a singleton delivery*

| **Table S1: Characteristics of obstetric patient population at each hospital for all delivery admissions between 01/2018 and 06/2023** | Academic Medical Center 1  n (%) | Suburban Hospital 1  n (%) | Suburban Hospital 2  n (%) | Suburban Hospital 3  n (%) | Five Exurban Community Hospitals  n (%) |
| --- | --- | --- | --- | --- | --- |
| **Total** | 62426 (100) | 8932 (100) | 14561 (100) | 7083 (100) | 11074 (100) |
| **Patient sociodemographic characteristics** | | | | | |
| **Maternal age (years)** |  |  |  |  |  |
| ≤ 19 | 538 (0.9) | 179 (2.0) | 157 (1.1) | 50 (0.7) | 259 (2.3) |
| 20-24 | 3479 (5.6) | 1092 (12.2) | 1045 (7.2) | 550 (7.8) | 1687 (15.2) |
| 25-29 | 8780 (14.1) | 2199 (24.6) | 3309 (22.7) | 1778 (25.1) | 3534 (31.9) |
| 30-34 | 26263 (42.1) | 3247 (36.4) | 6127 (42.1) | 3012 (42.5) | 3662 (33.1) |
| 35-39 | 19132 (30.6) | 1836 (20.6) | 3272 (22.5) | 1436 (20.3) | 1615 (14.6) |
| ≥ 40 | 4234 (6.8) | 379 (4.2) | 651 (4.5) | 257 (3.6) | 317 (2.9) |
| **Race/ethnicity (self-reported)** | | | | | |
| Non-Latinx White | 34386 (55.1) | 5149 (57.6) | 9621(66.1) | 5361(75.7) | 7636 (69.0) |
| Non-Latinx Black | 6278 (10.1) | 723 (8.1) | 666 (4.6) | 220 (3.1) | 854 (7.7) |
| Latinx | 9572 (15.3) | 1566 (17.5) | 1931 (13.3) | 942 (13.3) | 1510 (13.6) |
| Asian | 4508 (7.2) | 492 (5.5) | 1085 (7.5) | 168 (2.4) | 188 (1.7) |
| Other/unknown | 7682 (12.3) | 1002 (11.2) | 1258 (8.6) | 392 (5.5) | 996 (8.0) |
| **Medicaid** | 10721 (17.2) | 1835 (20.5) | 2697 (18.5) | 916 (12.9) | 3815 (34.5) |
| **Non-English language** | 2100 (3.4) | 384 (4.3) | 474 (3.3) | 115 (1.6) | 637 (5.8) |
| **Cesarean birth** | 16493 (26.4) | 2754 (30.8) | 4681(32.1) | 2256 (31.9) | 3193 (28.8) |
